# Highly Pathogenic Avian Influenza A(H5N1) Virus among Poultry, Ghana, 2015

**DOI:** 10.3201/eid2212.160639

**Published:** 2016-12

**Authors:** Ivy Asantewaa Asante, Stephanie Bertram, Joseph Awuni, Abraham Nii Okai Commey, Ben Aniwa, William Kwabena Ampofo, Gülsah Gabriel

**Affiliations:** Heinrich Pette Institute, Leibniz Institute for Experimental Virology, Hamburg, Germany (I.A. Asante, S. Bertram, G. Gabriel);; Noguchi Memorial Institute for Medical Research, University of Ghana, Accra, Ghana (I.A. Asante, W.K. Ampofo);; Center for Structural and Cellular Biology in Medicine at the University of Lübeck, Lübeck, Germany (S. Bertram, G. Gabriel);; Veterinary Services Directorate, Accra (J. Awuni, A.N.O. Commey, B. Aniwa)

**Keywords:** influenza, influenza in birds, highly pathogenic avian influenza, Africa, Ghana, poultry diseases, hemagglutinin avian influenza A virus, molecular typing, zoonoses, HPAI, H5N1, respiratory infections

**To the Editor:** Outbreaks of highly pathogenic avian influenza (HPAI) A(H5N1) virus among poultry were first reported in Africa in 2006, with initial reports from Nigeria (*1*). The virus then spread to several countries (e.g., Egypt, Côte d’Ivoire, Burkina Faso, Niger) in Africa, leading to large economic losses (*1*,*2*). In 2007, Ghana reported the first HPAI H5N1 cases among poultry in 3 regions: Greater Accra, Volta, and Brong Ahafo (*3*,*4*). The outbreak was contained by measures such as destruction of all birds on affected farms, disinfection of affected farms, and restricted movement of poultry and poultry products. Soon after containment, active influenza surveillance was initiated among birds, domestic poultry, and the human population throughout the country (*5*). Until 2015, no influenza-positive samples among birds had been detected in Ghana since the 2006–2007 outbreak. In January 2015, Nigeria resumed reporting HPAI H5N1–positive samples among poultry (*6*). One month later, HPAI H5N1–positive samples among chickens were confirmed in Burkina Faso (*7*). Then, in April 2015, chicken farmers in the Greater Accra region in Ghana reported a large number of deaths among domestic chicken flocks. Tracheal swabs collected from dead chickens and tested at the laboratories of the Veterinary Services Directorate in Accra, Ghana, confirmed the presence of HPAI H5N1 virus infection. By June 2015, the poultry populations in 5 of Ghana’s 10 regions were affected, leading to the death or culling of ≈100,000 poultry (*7*). Affected farms in Ghana (in the Greater Accra, Volta, and Ashanti regions) included medium-scale commercial farms with ≈30,000 chickens (broilers and layers) ranging from day-old chicks to layers >21 weeks of age; small-scale commercial bird farms with 200–1,000 chickens; and free-range local poultry of mixed species raised with low levels of biosafety. The death rate for chickens during the period of sample collection (April 13–June 11, 2015) was 17.6% (6,919 of 39,281 poultry died) (*7*). No direct links among farms were evident at this time. However, further spread in the Greater Accra region has been attributed to movement of live poultry. Outbreaks have been documented in live bird markets and backyard poultry, leading to a ban on movement of live poultry, feed, and equipment from affected regions. These counter-measures resulted in reduced incidence among poultry. For a lower-middle–income country like Ghana, such outbreaks are a major threat to food security and human health. We describe the outbreak strain found in Ghana during 2015 and its zoonotic potential. 

We obtained and analyzed sequences for the major viral genes involved in viral pathogenicity, such as the hemagglutinin (HA), polymerase basic protein 2 (PB2), nucleoprotein, and neuraminidase (NA) genes (Technical Appendix). Sequence analysis of HA revealed that the 2015 Ghana outbreak strain possessed a multibasic cleavage site (RERRRKR/GLF), which is common for the HPAI H5N1 virus (*8*). Substitutions (D94N, S133A, S155N, T156A [H5 numbering]) associated with increased virus binding to human-type α2-6-linked sialic acids were detected. Phylogenetic analysis confirmed that the Ghana outbreak strain belonged to clade 2.3.2.1c (Figure, panel A), as reported by the World Organisation for Animal Health in May 2015 (*7*). The strain clustered with contemporary viruses from Europe, Asia, the Middle East, and other West Africa countries (i.e., Burkina Faso, Côte d’Ivoire, Niger, and Nigeria). HA sequences of viruses isolated from 3 regions (Greater Accra, Volta, Ashanti) in Ghana were highly homologous, comparable to other Ghana viruses isolated in 2015 deposited in the Global Initiative on Sharing Avian Influenza Data (http://platform.gisaid.org) and GenBank databases (Figure, panel A). In total, 9 aa substitutions were detected in the Ghana strains as compared with the HPAI H5N1 virus that caused the 2015 outbreak in Nigeria (online Technical Appendix Table). Genetic analysis of PB2 showed that the Ghana viruses lacked the known human adaptive signatures E627K or D701N, which enable increased replication and virulence in the human host (*9*,*10*). Phylogenetic analysis revealed that PB2 of this virus clustered with H9N2 viruses isolated in Asia during 2007–2013 (Figure, panel B). Nucleoprotein sequences of the Ghana 2015 outbreak strain show 99% homology with the Nigeria 2015 outbreak strain. NA mutations known to reduce susceptibility to oseltamivir in NA H254Y were not observed.

**Figure:**
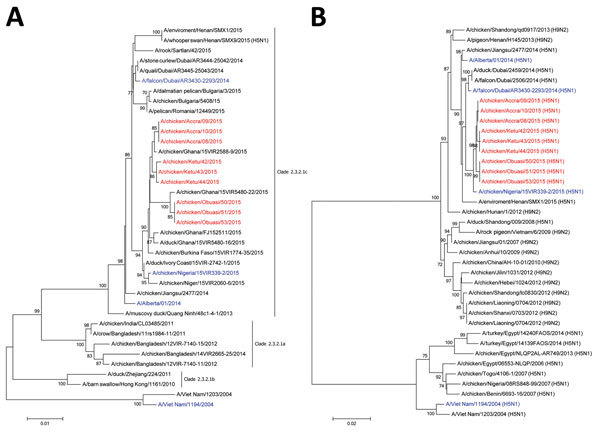
Phylogenetic analysis of highly pathogenic avian influenza A(H5N1) viruses isolated from poultry in Ghana in 2015: A) hemagglutinin; B) polymerase basic protein 2. Viruses sequenced for this study are in red and reference viruses are in blue; other sequences were downloaded from the Global Initiative on Sharing Avian Influenza Data (http://platform.gisaid.org) and GenBank databases. Evolutionary analyses were conducted with MEGA6 (http://www.megasoftware.net/). Bootstrap values >70% of 500 replicates are shown at the nodes. Scale bars indicate the number of nucleotide substitutions per site.

Our phylogenetic analysis suggests that the Ghana HPAI H5N1 strains belong to clade 2.3.2.1c, as was reported for the 2015 Nigeria outbreak. Because of the high homology (>90%) between the Ghana and Nigeria strains, the HPAI H5N1 Ghana outbreak strain likely originated in Nigeria (Figure, panel A). Migratory bird movements and human activities have been implicated in the virus’s introduction to the Africa continent. However, intercountry borders in West Africa are known to be porous because of frequent trading activities, possibly accounting for the spread of the virus in the subregion of West Africa.

Because the HPAI H5N1 virus of clade 2.3.2.1c has previously caused deaths in humans, the potential risk for transmission from infected poultry to humans is a major concern. Increased vigilance and rapid implementation of countermeasures are required to mitigate further virus adaptation and potential outbreaks among humans.

Technical Appendix Details of methods and of mutations in the highly pathogenic avian influenza A(H5N1) virus found in poultry in Ghana, 2015.
