# Supplementary material for: Highly Pathogenic Avian Influenza A(H5N1) Virus among Poultry, Ghana, 2015
Source: Emerg Infect Dis. 2016 Dec;22(12):2209–11. doi: 10.3201/eid2212.160639 (PMC5189147; doi:10.3201/eid2212.160639)
Supplement: Technical Appendix — Details of methods and of mutations in the highly pathogenic avian influenza A(H5N1) virus found in poultry in Ghana, 2015. [file 16-0639-Techapp-s1.pdf]

Article DOI: http://dx.doi.org/10.3201/eid2212.160639

Highly Pathogenic Avian Influenza A(H5N1) Virus among Poultry, Ghana, 2015

Technical Appendix

Detailed Methods

Lung samples were collected from dead birds (chickens, ducks, pigeons, and partridges) from farms in 3 affected regions (Greater Accra, Volta and Ashanti regions) in Ghana. Samples were frozen at –80°C in virus transport medium containing 2.5% veal infusion broth (Becton Dickinson, Franklin Lakes, NJ, USA), 0.5% bovine serum albumin (Sigma, St. Louis, MO, USA), 100mg/mL gentamicin (Gibco, Fisher Scientific, Pittsburgh, PA, USA), and 2 mg/mL fungizone (Hyclone Laboratory Inc., South Logan, UT, USA) and were shipped to the Heinrich Pette Institute, Leibniz Institute for Experimental Virology in Hamburg, Germany. Three lung tissue samples from chickens (layers >21 weeks of age) were randomly selected in each of the 3 affected regions and homogenized in phosphate-buffered saline. Virus-containing supernatants were used to inoculate 11-day-old embryonated specific-pathogen–free chicken embryos that were then incubated at 37°C for 48 hours. Infected chicken embryos were incubated at 4°C overnight and harvested the next day (*1*). Embryos were not alive at this point. Allantoic fluids were tested by using a standard hemagglutination assay, as previously described (*2*). Viral RNA isolated from positive allantoic fluids were subjected to Sanger sequencing (Seqlab Laboratories, Göttingen, Germany). Sequences were obtained for the hemagglutinin, basic polymerase protein 2, nucleoprotein, and neuraminidase genes. Sequences were assembled and analyzed by using Clone Manager 9 Professional Edition (Scientific and Educational Software, Denver, CO, USA). Phylogenetic analyses were performed by using sequences downloaded from the Global Initiative on Sharing All Influenza Data (http://platform.gisaid.org) and GenBank databases.

We compared sequences isolated from this study with virus strains that caused the 2015 highly pathogenic avian influenza A(H5N1) outbreak in Nigeria and to H5N1 strains obtained from the Global Initiative on Sharing All Influenza Data and GenBank databases (Technical

Appendix Table). Hemagglutinin from Ghana differed from the highly pathogenic avian influenza A(H5N1) virus from Nigeria by 9 aa changes. In the basic polymerase protein 2, from a cluster of 7 detected substitutions, 5 (L89V, G309D, T339K, R477G, K627E) were previously reported as increasing polymerase activity and virulence in mice (*1*). Comparing the strain from Ghana with the strain that caused the 2015 Nigeria outbreak revealed 2 aa changes in the nucleoprotein gene and 10 aa changes in the neuraminidase gene.

Technical Appendix Table. Mutations observed in highly pathogenic avian influenza A(H5N1) viruses in Ghana in 2015, compared with global strains and the 2015 outbreak strain from Nigeria (A/chicken/Nigeria/15VIR339–2/2015)*

	Details of mutations			
Protein	Regions	Previously published mutations	Functions of mutations	Literature sources
Polymerase basic protein 2	All regions	**L89V, G309D, T339K, R477G,** I495V, **K627E,** A676T	Enhanced polymerase activity; increased virulence in mice	Li et al., 2009 (*1*)
		I495A, A676M		
		M464L, V511I	Unknown	
	Accra, Ketu	M295V		
	Ketu	R17C		
	Obuasi	K197R		
Hemagglutinin	All regions	D94N	Increased virus binding to α2–6; enhanced virus fusion	Su et al., 2008 (*3*)
		S133A	Increased pseudovirus binding to α2–6	Yang et al., 2007 (*4*)
		S155N	Increased virus binding to α2–6	Wang et al., 2010 (*5*)
		T156A	Increased virus binding to α2–6; increased transmission in guinea pigs	Wang et al., 2010 (*5*); Gao et al., 2009 (*6*)
		S155N, T156A	Increased virus binding to α2–6	Wang et al., 2010 (*5*)
		323 to 330 (RERRRKRG)	Polybasic cleavage motif sequence required for high pathogenicity of H5N1 avian influenza viruses	Webster & Rott, 1987 (*7*); Horimoto & Kawaoka, 1994 (*8*); Schrauwen et al., 2012 (*9*); Sugitan et al., 2012 (*10*); Zhang et al., 2012 (*11*)
	All regions	T235P, I377V, K397R	Unknown	
	Accra	S356R		
	Ketu	T71S (G163S, T188A)†		
	Obuasi	K259R, K372R, N475D, M478I	Unknown	
Nucleoprotein	All regions	S450N	Unknown	
	Obuasi	Q398L		
Neuraminidase	All regions	E99Q	Unknown	
	Accra	I74V, R410Q		
	Accra, Ketu	S319L, S365C		
	Ketu	V243I, S430G		
	Obuasi	I143T, N202D, S319F		

*Bold fonts indicate that 5 mutations observed in sequences from Ghana are part of a group of 7 mutations that have been reported as enhancing polymerase activity and increasing virulence in mice (*1*).
†Mutations in parentheses were present in 1 of 3 samples from Ketu in the Volta Region of Ghana.

References

1. Li J, Ishaq M, Prudence M, Xi X, Hu T, Liu Q, et al. Single mutation at the amino acid position 627 of PB2 that leads to increased virulence of an H5N1 avian influenza virus during adaptation in mice can be compensated by multiple mutations at other sites of PB2. Virus Res. 2009;144:123–9. http://dx.doi.org/10.1016/j.virusres.2009.04.008

2. Kendal AP, Pereira MS, Skehel JJ, editors. Concepts and procedures for laboratory-based influenza surveillance. Atlanta: US Centers for Disease Control and Prevention; 1982.

3. Su Y, Yang H-Y, Zhang B-J, Jia H-L, Tien P. Analysis of a point mutation in H5N1 avian influenza virus hemagglutinin in relation to virus entry into live mammalian cells. Arch Virol. 2008;153:2253–61. http://dx.doi.org/10.1007/s00705-008-0255-y

4. Yang Z-Y, Wei C-J, Kong W-P, Wu L, Xu L, Smith DF, et al. Immunization by avian H5 influenza hemagglutinin mutants with altered receptor binding specificity. Science. 2007;317:825–8. http://dx.doi.org/10.1126/science.1135165

5. Wang W, Lu B, Zhou H, Suguitan AL Jr, Cheng X, Subbarao K, et al. Glycosylation at 158N of the hemagglutinin protein and receptor binding specificity synergistically affect the antigenicity and immunogenicity of a live attenuated H5N1 A/Vietnam/1203/2004 vaccine virus in ferrets. J Virol. 2010;84:6570–7. http://dx.doi.org/10.1128/JVI.00221-10

6. Gao Y, Zhang Y, Shinya K, Deng G, Jiang Y, Li Z, et al. Identification of amino acids in HA and PB2 critical for the transmission of H5N1 avian influenza viruses in a mammalian host. PLoS Pathog. 2009;5:e1000709. http://dx.doi.org/10.1371/journal.ppat.1000709

7. Webster RG, Rott R. Influenza virus A pathogenicity: the pivotal role of hemagglutinin. Cell. 1987;50:665–6. http://dx.doi.org/10.1016/0092-8674(87)90321-7

8. Horimoto T, Kawaoka Y. Reverse genetics provides direct evidence for a correlation of hemagglutinin cleavability and virulence of an avian influenza A virus. J Virol. 1994;68:3120–8.

9. Schrauwen EJA, Herfst S, Leijten LM, van Run P, Bestebroer TM, Linster M, et al. The multibasic cleavage site in H5N1 virus is critical for systemic spread along the olfactory and hematogenous routes in ferrets. J Virol. 2012;86:3975–84. http://dx.doi.org/10.1128/JVI.06828-11

10. Suguitan AL Jr, Matsuoka Y, Lau Y-F, Santos CP, Vogel L, Cheng LI, et al. The multibasic cleavage site of the hemagglutinin of highly pathogenic A/Vietnam/1203/2004 (H5N1) avian influenza virus acts as a virulence factor in a host-specific manner in mammals. J Virol. 2012;86:2706–14. http://dx.doi.org/10.1128/JVI.05546-11

11. Zhang Y, Sun Y, Sun H, Pu J, Bi Y, Shi Y, et al. A single amino acid at the hemagglutinin cleavage site contributes to the pathogenicity and neurovirulence of H5N1 influenza virus in mice. J Virol. 2012;86:6924–31. http://dx.doi.org/10.1128/JVI.07142-11
